# Rhode Island wildlife camera trap survey 2018 to 2023

**DOI:** 10.1002/ecy.70094

**Published:** 2025-05-08

**Authors:** Amy E. Mayer, Laken S. Ganoe, Charles Brown, Kylie Rezendes, Jessica Burr, Emerson Paton, Erin Wampole, Kimberly Rivera, Allison M. Stift, Krista L. Noe, Arianna E. Carey, Adriana Hughes, Thomas J. McGreevy, Brian D. Gerber

**Affiliations:** ^1^ Department of Natural Resources Science University of Rhode Island Kingston Rhode Island USA; ^2^ Rhode Island Department of Environmental Management, Division of Fish and Wildlife West Kingston Rhode Island USA; ^3^ Present address: University of Texas‐El Paso El Paso Texas USA; ^4^ Present address: Resources Management and Science, Yosemite National Park El Portal California USA; ^5^ Present address: Washington Department of Fish and Wildlife Ellensburg Washington USA; ^6^ Present address: Department of Conservation and Science Lincoln Park Zoo Chicago Illinois USA; ^7^ Present address: West Virginia Natural Heritage Program, West Virginia Division of Natural Resources Elkins West Virginia USA

**Keywords:** camera traps, mammals, multi‐season survey, Northeastern United States, occupancy modeling, Rhode Island, species distribution

## Abstract

Monitoring wildlife populations through the collection of abundance and distribution data across climatic seasons and multiple years is critical to understanding wildlife spatiotemporal dynamics. This is especially important in landscapes faced with natural and anthropogenic disturbances, which include the state of Rhode Island, USA. Rhode Island is the second most densely populated state in the United States, yet the landscape remains highly forested. Similar to many areas in the region, land cover change and conversion to non‐habitat cover types continue to be an issue as a result of increased anthropogenic disturbance, in addition to recent natural disturbance including forest structural changes from the spongy moth caterpillar ( *Lymantria dispar* ). These changes in land cover types and landscape patterns have the potential to positively or negatively affect wildlife communities, and thus, it is increasingly important to monitor wildlife populations. Camera traps provide an efficient way to inventory and monitor a large spatial area and record detections of a wide variety of terrestrial vertebrates. We began surveying the state of Rhode Island as part of a focal study on bobcats ( *Lynx rufus* , 2018–2020) and later fishers ( *Pekania pennanti* , 2020–2023) while documenting all species of terrestrial vertebrates detected at camera survey locations. We placed cameras in areas with land cover appropriate for the original target species—primarily forests and forested wetlands—and avoided placing cameras directly along hiking trails or roads. The state was divided into two sections—west and east—to maximize study area coverage with limited equipment. Cameras were deployed for at least six weeks in each survey period and section. In total, we monitored 249 survey sites in the state over 12 survey periods (six winter seasons, five summer seasons, and one spring season). We collected 244,013 unique detections from 39 terrestrial vertebrate species (25 mammal species, 13 bird species, and non‐personnel humans) throughout the study. These data provide spatial and temporal detection information that is useful for investigating the changes in wildlife populations over time and varying degrees of development through analyses including single species, multi‐species, dynamic, and diel occupancy modeling. Results of these analyses can be used to understand how a changing landscape impacts wildlife species. The data are openly available for reuse, and please cite this data paper when these data are used in other materials.

## CONFLICT OF INTEREST STATEMENT

The authors declare no conflicts of interest.

## Supporting information


Data S1.

## Data Availability

The dataset is available as Supporting Information to this paper (Data S1) and is also available on Zenodo at https://doi.org/10.5281/zenodo.10610602. The raw images used to compile the dataset are owned by the University of Rhode Island Department of Natural Resources Science and are available to qualified researchers by contacting the University of Rhode Island Department of Natural Resources Science Quest/Gerber Lab Manager (current email: agottfried@uri.edu) and requesting Rhode Island camera survey data from 2020 through 2023.

