# Supplementary material for: Rhode Island wildlife camera trap survey 2018 to 2023
Source: Ecology. 2025 May 8;106(5):e70094. doi: 10.1002/ecy.70094 (PMC12062420; doi:10.1002/ecy.70094)
Supplement: Supplementary file 1 — Data S1. [file ECY-106-e70094-s001.zip › Metadata_S1.pdf]

Data Paper published in *Ecology*, an Ecological Society of America journal

Metadata S1

Rhode Island wildlife camera trap survey 2018 to 2023

Amy E. Mayer, Laken S. Ganoe, Charles Brown, Kylie Rezendes, Jessica Burr, Emerson Paton, Erin Wampole, Kimberly Rivera, Allison M. Stift, Krista L. Noe, Arianna E. Carey, Adriana Hughes, Thomas J. McGreevy Jr., Brian D. Gerber

INTRODUCTION

Wildlife populations can fluctuate over time in response to natural processes and disturbances (Holt and Keitt 2000, Bellard et al. 2012). Monitoring changes in the distribution of wildlife species over time is critical for effective management and conservation of wildlife, particularly as many landscapes are increasingly affected by both natural and anthropogenic change (Mackenzie et al. 2006, Westgate et al. 2013, Farris et al. 2017). Collecting data systematically and consistently over longer periods of time provides fine-scale information that can be paired with landscape information to understand how changes to the landscapes affect a species' abundance and distribution (McRae et al. 2008, Clutton-Brock and Sheldon 2010).

The United States has been in a period of landscape change and loss due to urbanization and an expansion of exurban areas (Brown et al. 2005, Hansen et al. 2005, Theobald 2010). Many wildlife species have shown negative responses to these landscape changes (Crooks 2002, Moss et al. 2016, Wait et al. 2018), while others have demonstrated the ability to adapt to increased levels of human development (Gompper 2002, Prange and Gehrt 2004, Wang et al. 2015, Mayer et al. 2023). As these landscape changes become more common and widespread, understanding how a species adapts to both natural and anthropogenic landscape changes is critical for conserving and managing wildlife populations.

In recent years, with improvements in technology and lower costs, camera traps have become a useful tool for non-invasively monitoring a wide variety of wildlife species (Kucera and Barrett 2011, Burton et al. 2015). By passively recording spatial and temporal detection and occurrence data, scientists can understand where and when a species is occurring on the landscape, and how the occurrence varies over time.

Here, we present a dataset of camera trap survey data from January 2018 to April 2023 at 249 survey locations in the state of Rhode Island (Figure 1). This dataset includes detections of 25 mammal species (Table 1) across 12 survey seasons and 138,148 trap nights (Table 2). Due to the relatively high amount of forest cover, and the high anthropogenic footprint, Rhode Island is an interesting study area to investigate the effect of human development on a community of wildlife. Additionally, our study began in the years following large-scale natural disturbances due to spongy moth (*Lymantria dispar*) caterpillar outbreak (2015-2017) where many areas suffered severe defoliation followed by tree mortality (Pasquarella et al. 2018). The data presented can be used to conduct single season and dynamic occupancy analyses, investigate species distribution, activity patterns, and interactions between species both in a single survey season and across multiple temporal scales. (eg diel, seasonal, and annual variation.) Additionally, the timing and duration of the survey, and the scale and locations of the survey sites will allow researchers to investigate the long-term effects of both natural and anthropogenic disturbance on a wide variety of terrestrial vertebrates.

While survey locations and survey effort are documented thoroughly and accurately in the datasets, users should be aware of variations in survey methodology, particularly during the first three field seasons, and we suggest users of this dataset account for variation in survey effort when conducting future analyses. Notably, while the number of survey locations and cameras deployed varied during the course of the study, a core set of 100 survey locations was consistent for nine of the 12 survey seasons, and the effort at survey sites (i.e. number of cameras at a location) was consistent with two cameras per site for eight of the 12 survey seasons, allowing for the analysis of multiple seasons of consistent location and effort.

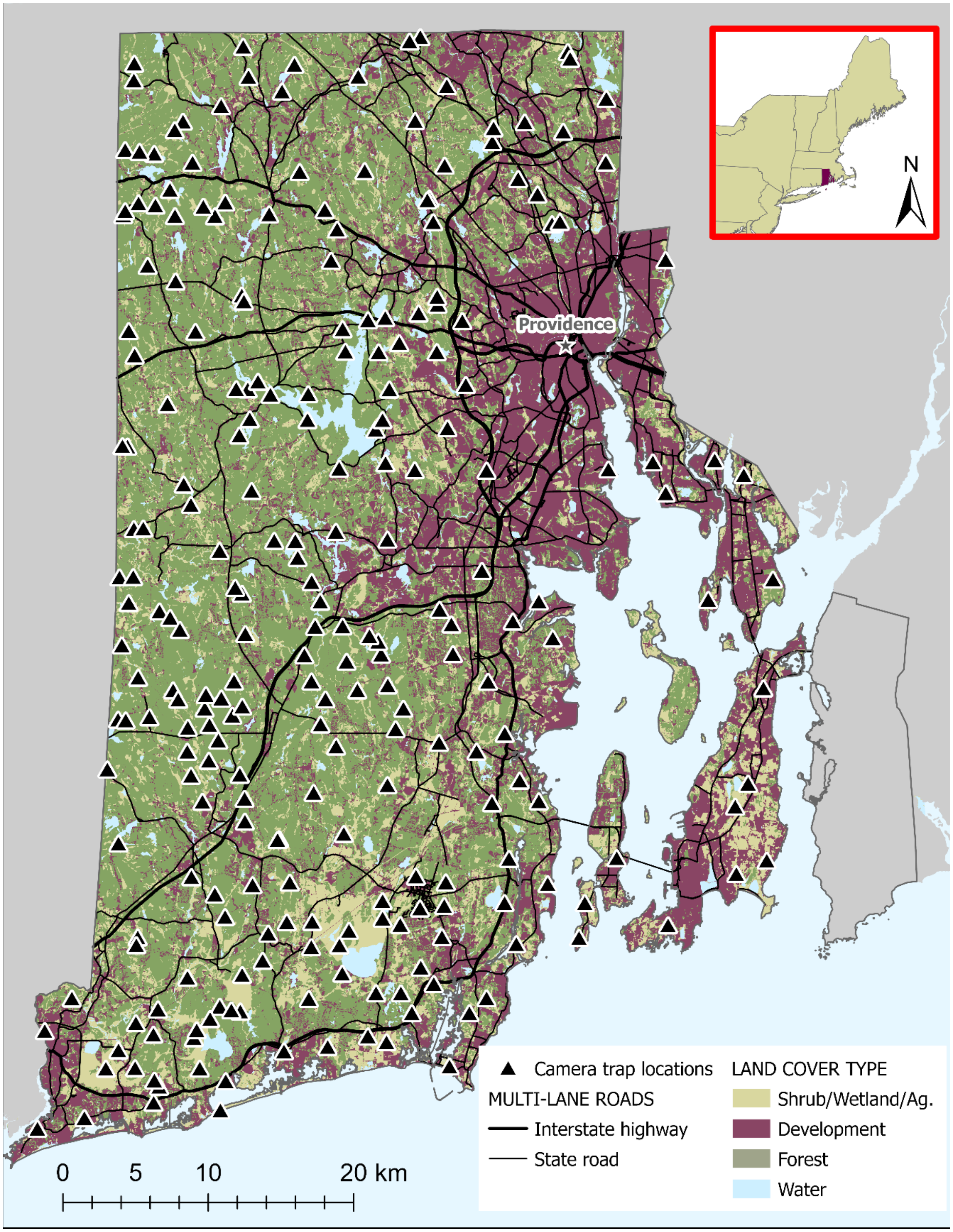

Figure 1: Location of survey sites Rhode Island January 2018 to April 2023. Our study area

included all five counties in Rhode Island, USA (Washington, Kent, Providence, Bristol, and Newport).

Table 1: Number of independent detections of species by survey season. Winter surveys took place between November and March, Summer surveys took place between May and September. The only Spring season took place in March and April on a small number of survey sites on islands that had not been previously surveyed. Detections here are considered independent if there is more than one individual captured in a photograph, or if >20 minutes has elapsed since the last detection of the same species at the same camera trap. Species were identified to the lowest taxonomic unit when possible.

Species	Winter 2018	Summer 2018	Winter 2019	Summer 2019	Winter 2020	Summer 2020	Winter 2021	Summer 2021	Winter 2022	Spring 2022	Summer 2022	Winter 2023
Canis familiaris	72	34	227	155	525	78	292	60	191	3	47	222
Canis latrans	161	182	433	317	607	521	925	675	1,092	25	693	568
Castor canadensis	-	-	-	-	-	-	-	6	9	-	5	16
Didelphis virginiana	4	591	39	447	29	476	78	1,036	233	23	1,217	111
Erethizon dorsatum	-	-	-	-	-	-	1	-	-	-	-	-
Felis catus	8	29	5	30	8	35	53	56	53	4	41	3
Glaucomys volans	1	7	5	46	15	13	335	217	1,751	-	274	70
Homo sapiens	-	8	166	93	531	141	769	147	259	16	79	340
Lontra canadensis	-	-	4	5	3	-	39	22	13	1	42	17
Lynx rufus	42	16	96	52	77	32	112	70	110	-	131	118
Marmota monax	-	17	3	8	-	25	-	56	-	1	80	2
Meleagris gallopavo	62	115	654	563	238	158	1,017	390	386	5	537	727
Mephitis mephitis	12	35	18	48	35	48	67	144	36	5	216	117

Mustela frenata	10	23	8	38	12	58	71	481	136	-	703	115
Neovison vison	1	4	1	8	10	16	73	74	63	3	51	40
Odocoileus virginianus	665	1,244	1,374	2,217	1,593	2,414	5,251	5,202	4,293	106	4,728	1,973
Ondatra zibethicus	-	-	-	1	-	-	-	-	20	3	4	5
Pekania pennanti	198	231	387	317	554	259	779	338	683	-	246	215
Procyon lotor	172	578	287	873	558	1,003	1,532	2,270	2,137	95	2,162	915
Rodentia sp.	10	94	94	420	397	343	7,209	14,266	34,275	608	12,685	1,020
Sciurus carolinensis	99	782	529	2,667	931	1,369	6,652	8,554	13,274	268	10,844	2,034
Sylvilagus floridanus	129	87	408	212	627	323	1,189	635	1,148	351	565	1,422
Tamias striatus	1	107	7	133	22	510	854	3,178	902	-	4,203	66
Tamiasciurus hudsonicus	53	121	66	365	197	352	1,131	2,408	2,516	-	2,554	373
Urocyon cinereoargenteus	24	27	164	50	142	73	214	136	178	-	158	77
Ursus americanus	-	1	-	-	-	2	-	2	-	-	5	-
Vulpes vulpes	46	50	105	126	173	69	232	200	518	-	267	198
Aix sponsa	-	-	1	-	9	-	-	-	-	-	-	-
Ardea herodias	-	-	-	-	19	-	1	3	2	-	-	25
Aves sp.	24	387	146	649	208	1,062	1,218	5,579	2,455	132	6,383	827
Buteo jamaicensis	-	-	3	8	3	8	-	-	-	-	-	-
Buteo platypterus	-	-	-	3	-	-	-	-	-	-	-	-

Cathartes aura	-	2	5	7	1	1	-	-	-	-	-	-
Corvus brachyrhynchos	-	5	-	8	12	-	-	-	-	-	-	-
Dryocopus pileatus	-	1	-	1	-	6	-	-	-	-	-	-
Phasianus colchicus	-	-	-	-	3	-	-	-	12	-	-	1
Picidae sp.	-	1	3	2	5	-	-	-	-	-	-	-
Scolopax minor	-	-	-	1	9	2	9	13	14	-	10	26
Strix varia	8	4	4	2	2	3	3	15	9	-	14	16

Table 2: Camera trapping effort by season. Winter surveys took place between November and March, Summer surveys took place between May and September. The only Spring season took place in March and April on a small number of survey sites on islands that had not been previously surveyed.

Season	# Survey Locations	# Cameras (Total)	Total Trap Nights	Mean # Days Camera Active (Range)
Winter 2018	40	40	3,411	85.28 (63 – 109)
Summer 2018	100	100	4,485	44.85 (13 – 58)
Winter 2019	20	50	7,128	146.13 (140 – 151)
Summer 2019	100	200	7,802	41.02 (27 – 52)
Winter 2020	100	200	9,462	49.37 (35 – 60)
Summer 2020	100	200	8,706	45.38 (32 – 49)
Winter 2021	200	400	21,536	56.17 (33 – 71)
Summer 2021	240	480	20,219	44.81 (37 – 55)
Winter 2022	239	478	23,277	51.36 (6.5 – 88.5)
Spring 2022	9	18	483	36.5 (4 – 44)
Summer 2022	240	480	20,177	44.49 (5 – 54)
Winter 2023	100	200	11,462	59.64 (42 – 86)

CLASS I. DATA SET DESCRIPTORS

A. Data set identity: Camera trap data from survey of conservation land in Rhode Island collected seasonally from 2018 to 2023.

B. Data set identification code:

RI_CameraSurvey_Detections.csv

RI_CameraSurvey_Deployments.csv

C. Data set description

1.) Originators:

Amy E. Mayer

Department of Natural Resources Science, University of Rhode Island, Kingston, RI, 02881, USA

Laken S. Ganoe

Department of Natural Resources Science, University of Rhode Island, Kingston, RI, 02881, USA

Charles Brown

Rhode Island Department of Environmental Management, Division of Fish and Wildlife, West Kingston, RI 02892, USA

Thomas J. McGreevy, Jr.

Department of Natural Resources Science, University of Rhode Island, Kingston, RI, 02881, USA

Brian D. Gerber

Department of Natural Resources Science, University of Rhode Island, Kingston, RI, 02881, USA

2.) Abstract: Monitoring wildlife populations through the collection of abundance and distribution data across climatic seasons and multiple years is critical to understand wildlife spatio-temporal dynamics. This is especially important in landscapes faced with natural and anthropogenic disturbances, which includes the state of Rhode Island, USA. Rhode Island is the second most densely populated state in the United States, yet the landscape remains highly forested. Similar to many areas in the region, landcover change

and conversion to non-habitat cover types continues to be an issue as a result of increased anthropogenic disturbance, in addition to recent natural disturbance including forest structural changes from the spongy moth caterpillar (*Lymantria dispar*). These changes in landcover types and landscape patterns have the potential to positively or negatively affect wildlife communities, and thus it is increasingly important to monitor wildlife populations. Camera traps provide an efficient way to inventory and monitor a large spatial area and record detections of a wide variety of terrestrial vertebrates. We began surveying the state of Rhode Island as part of a focal study on bobcats (*Lynx rufus*, 2018 - 2020), and later fishers (*Pekania pennanti*, 2020 - 2023) while documenting all species of terrestrial vertebrates detected at camera survey locations. We placed cameras in areas with land cover appropriate for the original target species – primarily forests and forested wetlands – and avoided placing cameras directly along hiking trails or roads. The state was divided into two sections – west and east – to maximize study area coverage with limited equipment. Cameras were deployed for at least six weeks in each survey period and section. In total, we monitored 249 survey sites in the state over 12 survey periods (six winter seasons, five summer seasons, and one spring season). We collected 244,013 unique detections from 39 terrestrial vertebrate species (25 mammal species, 13 bird species, and non-personnel humans) throughout the study. These data provide spatial and temporal detection information that is useful for investigating the changes in wildlife populations over time and varying degrees of development through analyses including single species, multi-species, dynamic, and diel occupancy modeling. Results of these analyses can be used to understand how a changing landscape impacts wildlife species.

The data are openly available for reuse and please cite this data paper when these data are used in other materials.

D. Key words/phrases: Camera traps, mammals, multi-season survey, Northeast United States, occupancy modeling, Rhode Island, species distribution.

CLASS II. RESEARCH ORIGIN DESCRIPTORS

A. Overall project description:

1.) Identity: Rhode Island Wildlife Camera Trap Survey 2018 to 2023

2.) Originators(s): Amy E. Mayer, Laken S. Ganoe, Charles Brown, Thomas J. McGreevy Jr., and Brian D. Gerber coordinated the survey methods and acquisition of data.

3.) Period of study: January 2018 to April 2023

4.) Objectives: The aim of the study was to document and monitor wildlife populations across Rhode Island. The study began in winter 2018 with the goal of using camera traps to analyze the occupancy and distribution of bobcats in the state of Rhode Island. In winter 2021, we added additional survey sites to the existing locations where the main objective was to evaluate the occupancy of fisher in the state of Rhode Island.

5.) Sources of funding: Rhode Island Department of Environmental Management Division of Fish and Wildlife, Wildlife Restoration Grants administered by the U.S. Fish and Wildlife Service, Wildlife and Sport Fish Restoration Program projects W23R and F19F01093.

B. Specific subproject description

1.) Site description

a. Site type: All surveys were conducted in Rhode Island on publicly accessible properties or areas designated as conservation land. This included state-owned management areas and parks, land trusts, municipal parks and forests, and non-profit land conservation areas. The majority of individual cameras were located in forests (deciduous, softwood, or mixed; n = 240). Others were placed in wetlands (n = 4), brushland (n = 2), or other land cover types (n = 3; Figure 1).

b. Geography: The surveys were conducted in all five counties in Rhode Island, USA (Washington, Kent, Providence, Newport, and Bristol) (Figure 1). Survey site coordinates can be found in the camera deployment table.

c. Habitat: Rhode Island has a high percentage of forest cover as well as a high anthropogenic footprint. Survey sites were primarily located in forested areas with varying levels of anthropogenic development surrounding the survey sites (Figure 1).

2.) Sampling Design and Research Methods

We surveyed state, municipal and non-governmental conservation land in Rhode Island by deploying motion-triggered trail cameras at survey sites across multiple field seasons. To establish the survey locations, a 1 km^2 grid was laid over the state of Rhode Island. Grid cells were considered available for a survey site if they contained at least 4 ha of conservation land (RIGIS 2018a, 2018b). Sites were selected using stratified random sampling to ensure the land cover types in the surveyed areas, primarily forests and forested wetlands, were surveyed proportional to the overall land covers available in the state. Areas with high proportions of development were excluded, specifically the highly urban areas in and around Providence (Figure 1), because grid cells in these areas did not meet the minimum requirement of 4 ha of conservation land, and thus areas of

high development were under-represented in surveyed sites (Figure 2). Within a selected grid cell, one survey location was randomly placed within a parcel where both the land was designated for conservation and permission had been granted by the landowner. In the field, the camera was placed at an appropriate location (i.e. away from roads, heavily traveled hiking trails, and in areas with suitable cover for the target species) within 100m of this randomly generated survey point.

During survey season 1 (winter 2018), 40 survey locations were chosen in the southern portion of the state. At each survey site, one Browning Strike Force Pro XD (Browning, Morgan, UT, USA) or Bushnell Trophy Cam (Bushnell Outdoor Products, Overland Park, KS, USA) motion-triggered trail camera was placed 0.5 to 1 m above and angled parallel to the ground, facing north. Vegetation directly in the field of view of the camera was trimmed to improve visibility and reduce false triggers. Both camera models used in this study used red glow infrared flash for night photos, and had average reported detection distances of 80 feet and average trigger speed is 0.15 seconds. Cameras were set to take a burst of three photos when triggered with a 10 second rest period between triggers. A commercial scent lure ("Caven's Gusto"; Minnesota Trapline Products, Pennock, MN, USA) was applied to a tree approximately 2 m in front of the camera and 2 m off the ground at camera deployment to increase the chance of animals walking in front of the camera. In survey season 2 (summer 2018), the survey was expanded to include 100 survey locations statewide. The new camera locations were selected and set-up as above. In response to low detection rates of the initial target species (bobcat, *Lynx rufus*), in survey season 3 (winter 2019) we added additional cameras to a subset of locations to determine if additional cameras at a site would increase detection rates. We

surveyed 20 of the original survey sites from survey season 1 and deployed one or two additional cameras within 1 ha of the original camera location following the same set-up protocol as above. Ten sites had a total of two cameras each and 10 sites had a total of three cameras each. Due to the proximity of the additional cameras to the original camera location, there were no differences in landcover type between the cameras at a survey site. We found that adding one additional camera sufficiently increased the probability of detecting the initial target species at a survey site, so for all remaining survey seasons all survey sites were set up with two cameras that were an average of 55 m apart (range: 20 m – 130 m).

In survey season 7 (winter 2021) we added 100 additional survey sites for a total of 200 sites with two cameras each. In survey season 8 (summer 2021) we expanded the survey again and added 40 additional locations with two cameras each for a total of 240 survey sites (Figure 1). The final average distance between primary survey sites was 1,794 m (range: 135 m – 7,729 m). Several mammal species, including bobcat and fisher, have not been documented on Aquidneck and Conanicut Islands in Narragansett Bay. To confirm this, during the spring of 2022 we deployed 18 cameras at nine survey locations on Aquidneck and Conanicut Islands using the same protocols as above. The final survey season, winter 2023, the survey sites were reduced back to the initial 100 statewide survey sites.

Cameras were deployed for at least six weeks during each survey season. We used the camera trap database 'Camelot' (Hendry and Mann 2018) to record all camera deployment data, and to organize, process and add identifications to each photo collected in the survey. If cameras malfunctioned for a portion of the deployment and/or were

replaced mid-deployment, a new start date was recorded in the database at the time the camera malfunction was resolved to accurately reflect the amount of time cameras were operational. We added the number of individuals and the species identification to each photo when possible. If an animal could not be identified to species, we applied the lowest possible taxonomic unit to the identification of that photo. All detections and camera deployment data were exported to .csv format files directly from the Camelot database. We included all detections of wildlife species, domestic animals (dogs and cats), and humans other than project personnel in our final dataset. While medium to large mammals are detected most reliably by the camera traps, the dataset also includes detections of small mammals, including rodents (identified as Rodent sp.), small birds (identified as Aves sp.), birds of prey, and wild turkeys (Figures 3 and 4).

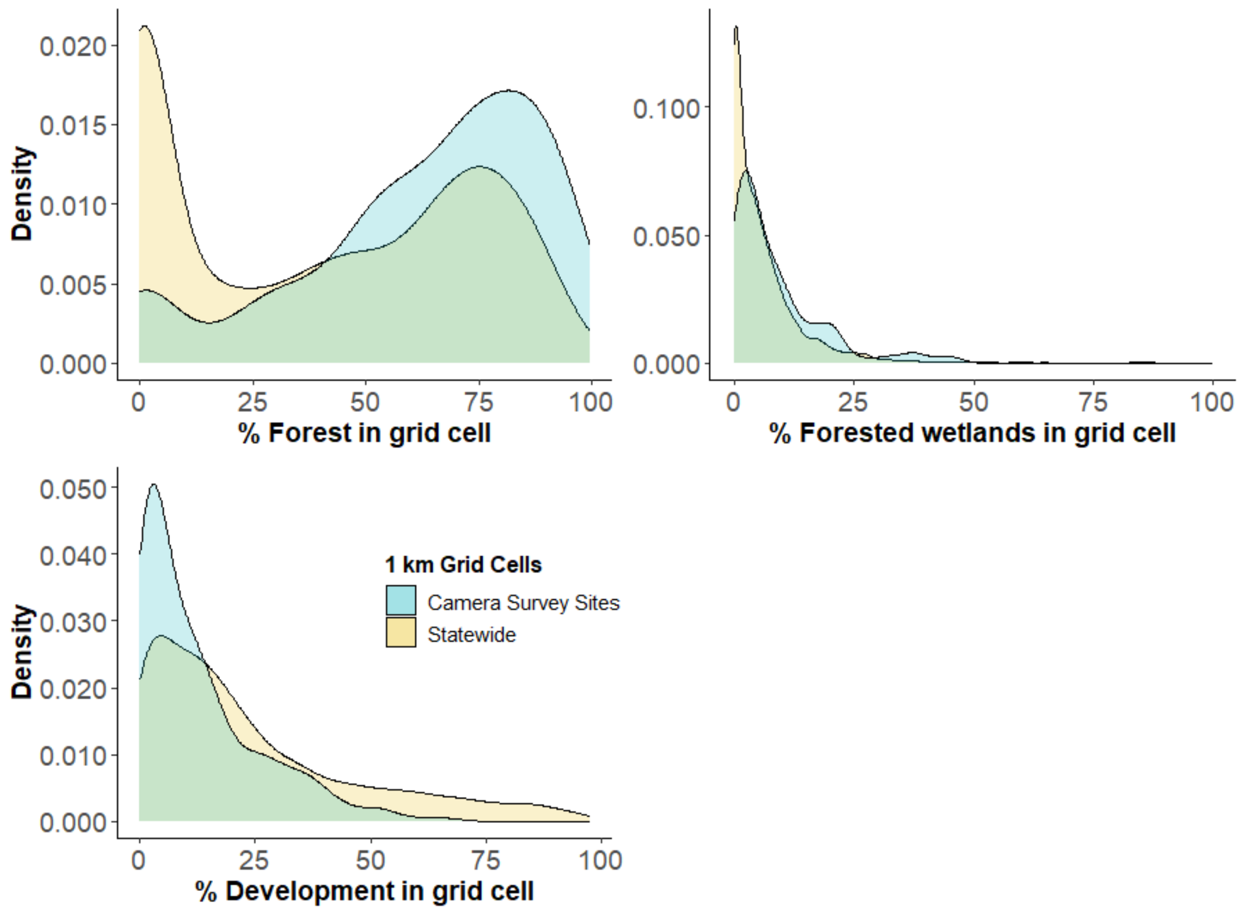

Figure 2: Comparison of a selection of land cover types that were surveyed versus what was available to be surveyed across the entire study area. Higher proportions of forests and forested wetlands were well represented at survey sites proportional to the study area; however, areas of high development were not represented in the survey.

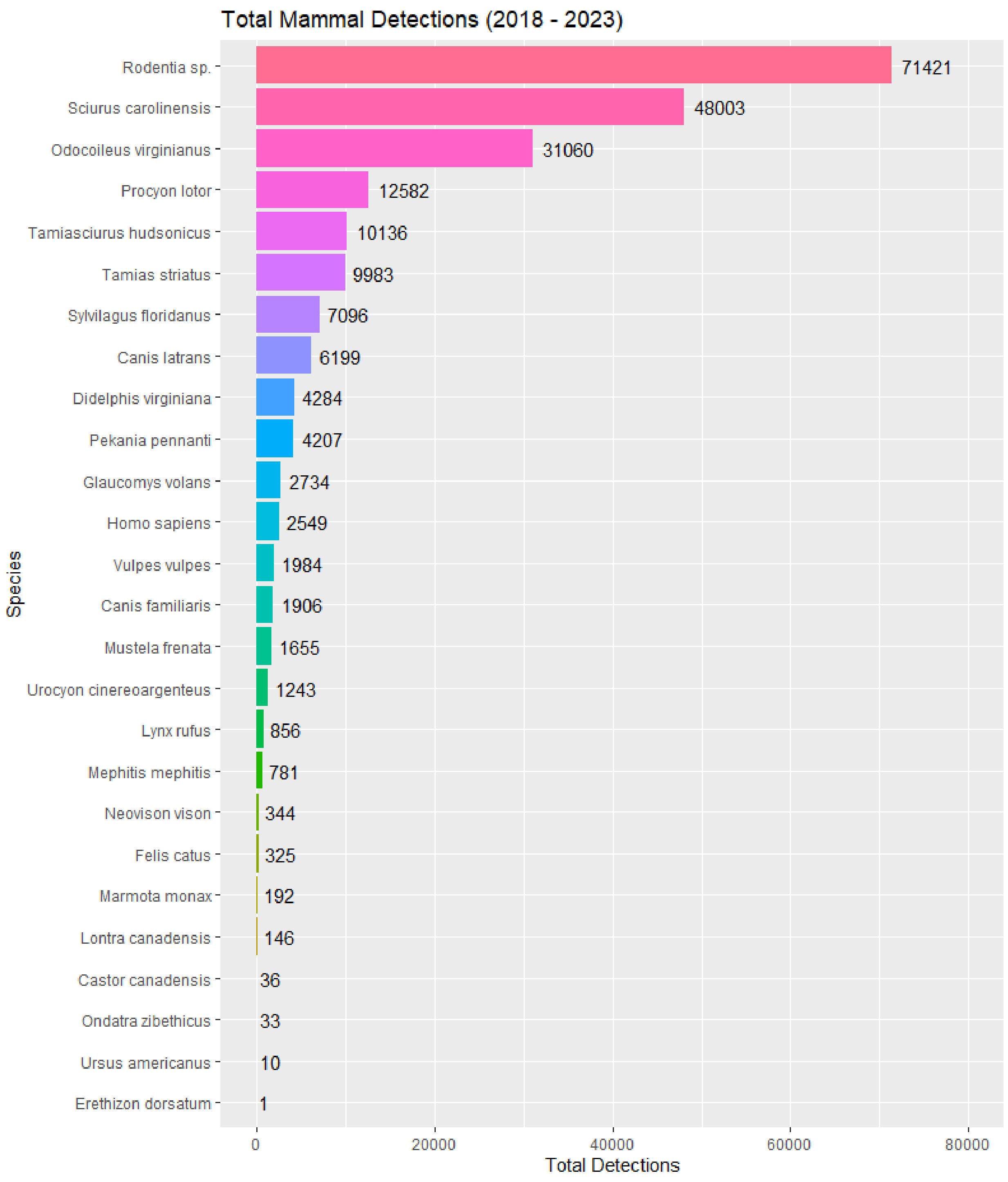

Figure 3: Total number of independent detections of all mammal species across all survey seasons. Detections here are considered independent if there is more than one individual captured in a photograph, or if >20 minutes has elapsed since the last detection of the same species at the

same camera trap. When mammals could not be identified to *Genus* and *species*, we identified the detection to the lowest possible taxonomic unit.

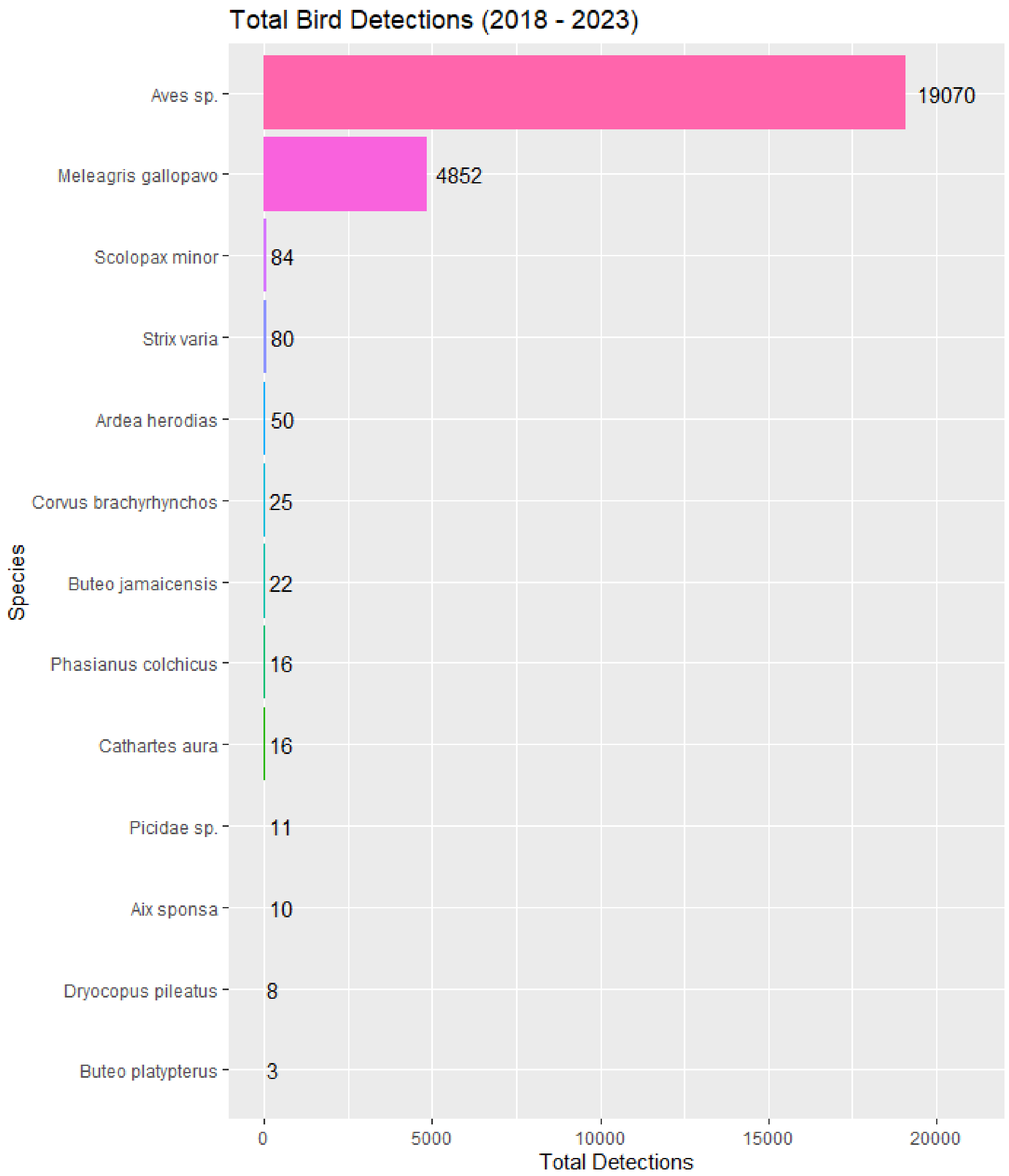

Figure 4: Total number of independent detections of all bird species across all survey seasons. Detections here are considered independent if there is more than one individual captured in a photograph, or if >20 minutes has elapsed since the last detection of the same species at the same camera trap. When birds could not be identified to *Genus* and *species*, we identified the detection to the lowest possible taxonomic unit.

a. Instrumentation: Browning Strike Force Pro XD (Browning, Morgan, UT, USA); Bushnell Trophy Cam (Bushnell Outdoor Products, Overland Park, KS, USA).

b. Taxonomy and systematics: We followed taxonomy and naming conventions of the Integrated Taxonomic Information System online database (ITIS; http://www.itis.gov)

3.) Project personnel: In addition to Project Originators/Co-Authors, key personnel include seasonal technicians and undergraduate assistants.

Seasonal technicians: Jess Burr (2022), Emerson Paton (2021), Kylie Rezendes (2020-2022)

Undergraduate assistants: Jake Brown (2020), Arianna Carey (2018-2020), Ryan Healey (2019-2020), Adriana Hughes (2018), Tess Kostisin (2019), Krista Noe (2018-2019), Ian O'Hara (2018), Daniel Reisch (2020), Kylie Rezendes (2019), and Allie Stift (2019-2020).

C. Data Limitations

The first three survey seasons (winter 2018, summer 2018, winter 2019) were treated as pilot seasons where we were finalizing methodology and site selection. As such, care should be taken to account for survey effort (available in the deployment table) during the first three seasons when comparing results from these seasons across time. There were no tagged individuals during this study, and all of the data presented here is strictly

detection/non-detection for a given location and time. Therefore, using this data to estimate abundance or population density requires additional modeling which may be challenging and requires careful consideration of model assumptions (Gilbert et al. 2021). While we included detections of small rodents and birds, camera traps often do not capture these wildlife species consistently as they don't always trigger the motion sensors due to their small size and fast movements. Additionally, identifying small rodents and song birds beyond taxonomic Order or Class with certainty can be very difficult, particularly in nighttime detections when the images are in greyscale. To standardize detections avoid misidentification we tagged small rodents and birds as Rodentia sp. or Aves sp., respectively.

CLASS III. DATA SET STATUS AND ACCESSIBILITY

A. Status

1.) Latest update: 17 December 2024

2.) Latest archive date: 17 December 2024

3.) Metadata status: 17 December 2024

4.) Data verification: Data was reviewed for correct species identification and spatial and temporal accuracy prior to archiving. At the conclusion of data collection and processing, a random subset of 10% of all collected images were reviewed by trained technicians to ensure correct identification. Additionally, all images that were initially tagged as a species that may be commonly misidentified including coyote (*Canis latrans*), red fox (*Vulpes vulpes*), gray fox (*Urocyon cinereoargenteus*), fisher (*Pekania pennanti*), mink (*Neovison vison*), long-tailed weasel (*Mustela frenata*), and bobcat (*Lynx rufus*) were reviewed by two authors (A. Mayer and L. Ganoe) for species verification.

B. Accessibility

1.) Storage location and medium: In addition to this publication, data are available for download directly from Zenodo at: https://doi.org/10.5281/zenodo.10610602. This consists of 2 datasets (deployments and detections) in .csv format. The raw images used to compile the available datasets are owned by the University of Rhode Island Department of Natural Resources Science and available to qualified researchers by contacting the URI Department of Natural Resources Science, Quest/Gerber Lab Manager (current email: agottfried@uri.edu) and requesting Rhode Island camera survey data from 2020 through 2023.

2.) Contact persons: Amy E. Mayer, Department of Natural Resources Science, University of Rhode Island, 1 Greenhouse Road, Kingston, RI 02881, USA. Email: agottfried@uri.edu

3.) Copyright restrictions: None

4.) Proprietary restrictions: Please cite this paper when using the data for publication

5.) Costs: None

CLASS IV. DATA STRUCTURAL DESCRIPTORS

A. Data set file

1.) Identity: DataS1.zip contains:

RI_CameraSurvey_Deployments.csv

RI_CameraSurvey_Detections.csv

2.) Size: Total: 12.1 MB (zip)

RI_CameraSurvey_Deployments.csv: 2,982 rows, 8 columns, 273.9 KB

RI_CameraSurvey_Detections.csv: 1,385,615 rows, 15 columns 245.2 MB

3.) Format and storage mode: comma-separated values (.csv)

4.) Header information: See column description in Section B

5.) Alphanumeric attributes: Mixed

6.) Special characters/fields: None

7.) Authentication procedures: None

B. Variable information

Table 3: Variable information for camera deployment data.

Variable	Type	Definition
YearSeason	One of 12 unique text strings representing a survey season	Combination of season and year. Summer (s), winter (w), or spring (sp) paired with the last two digits of the calendar year (18-23).
Primary.Site.ID	One of 265 unique text strings representing a survey location	Unique identification assigned to each primary survey site location. Beginning in season 'w19', multiple cameras were placed within a primary survey site.
Trap.Station.Name	One of 524 unique text strings representing the camera trap station within a primary survey site	Unique identification assigned to the camera trap location within a primary survey site. Trap.Station.Name and Primary.Site.ID may be the same if the survey site was established prior to season 'w19' when only a single camera was used per survey site.
Camera.Name	One of 373 unique text strings representing the identification assigned to a specific camera	Text string assigned to a particular trail camera to track inventory. Cameras may be used multiple times during a survey season, but the deployment dates do not overlap.
Setup.Date	Date camera was set up in the format: "YYYY-MM-DD"	First date of the camera trap deployment. There may be

		multiple setup dates for a camera trap within a survey season if the camera malfunctioned and was reset during a deployment
Retrieval.Date	Date camera and data was retrieved in the format: "YYYY-MM-DD"	Date of the camera trap/data retrieval. There may be multiple retrieval dates for a camera trap within a survey if the camera malfunctioned and was reset during a deployment.
Latitude	Double precision vector	Latitude value of a specific camera trap at a station and deployment. Coordinates are provided in decimal degrees (WGS 84).
Longitude	Double precision vector	Longitude value of a specific camera trap at a station and deployment. Coordinates are provided in decimal degrees (WGS 84).

Table 4: Variable information for species detection data.

Variable	Type	Definition
YearSeason	One of 12 unique text strings representing a survey season	Combination of season and year. Summer (s), winter (w), or spring (sp) paired with the last two digits of the calendar year (18-23).
Primary.Site.ID	One of 265 unique text strings representing a survey location	Unique identification assigned to each primary survey site location. Beginning in season 'w19', multiple cameras were placed within a primary survey site.
Trap.Station.Name	One of 524 unique text strings representing the camera trap station within a primary survey site	Unique identification assigned to the camera trap location within a primary survey site. Trap.Station.Name and Primary.Site.ID may be the same if the survey site was established prior to season

		'w19' when only a single camera was used per survey site.
Camera.Name	One of 373 unique text strings representing the identification assigned to a specific camera	Text string assigned to a particular trail camera to track inventory. Cameras may be used multiple times during a survey season, but the deployment dates do not overlap.
DateTime	Date and time stamp for an observation in the format "YYYY-MM-DD HH:MM:SS"	Timestamp of each tagged image. Timestamps were recorded in Eastern Standard Time (EST). Timestamp metadata was manually adjusted to account for daylight savings time when necessary.
Date	Date of an observation in the format "YYYY-MM-DD"	Date of each tagged image.
Time	Time of an observation in the format "HH:MM:SS"	Time of each tagged image.
Common.Name	Text string with the common name	Common name of the species assigned to each detection.
Class	Text string with the taxonomic class	Taxonomic class assigned to each detection. All detections are either in class Mammalia or Aves.
Order	Text string with the taxonomic order	Taxonomic order assigned to each detection. If the animal could not be identified beyond taxonomic class, the order field will contain 'NA.'
Family	Text string of the taxonomic family	Taxonomic family assigned to each detection. If the animal could not be identified beyond order and/or class, the family field will contain 'NA.'
Genus	Text string of the taxonomic genus	Taxonomic genus assigned to each detection. If the animal could not be

		identified beyond order, class, and/or family, the genus field will contain 'NA.'
Species	Text string of the taxonomic specific epithet	Taxonomic specific epithet assigned to each detection. If the animal could not be identified beyond order, class, family, and/or genus, the species field will contain 'NA.'
Genus.and.Species	Text string in the format: "*Genus species*" for identifiable species. If species cannot be determined, the format may be "*Order* sp.", "*Family* sp.", or "*Genus* sp."	The binomial scientific name, or lowest possible taxonomy, of species detected.
Sighting.Quantity	Integer of total number of individuals present in the image	The number of individuals of each species visible in each image.

CLASS V. SUPPLEMENTAL DESCRIPTORS

A. Data acquisition

1.) Data forms or acquisition methods: NA

2.) Location of completed data forms: NA

3.) Data entry verification procedures: NA

B. Quality assurance/quality control procedures: Detection species identifications were screened for quality assurance as outlined in Section IIIa. Location data was screened by mapping coordinates and visually confirming camera trap location.

C. Related materials: NA

D. Computer programs and data-processing algorithms: Raw photo data was processed, cataloged and identified using the camera trap database 'Camelot' (Hendry and Mann 2018).

E. Archiving

1.) Archival procedures: All raw photographs from camera traps are stored on physical external hard drives and archived in web-based cloud storage. Raw images are available upon request.

F. Publications and results: The following publications used subsets of the data included in this publication for analysis:

1. Ganoe, L. S., A. E. Mayer, C. Brown, B. D. Gerber. 2024. Mesocarnivore sensitivity to natural and anthropogenic disturbance leads to declines in occurrence and concern for species persistence. Ecology and Evolution 14(7).
2. Twining, J. P., et al. 2024. Using global remote camera data of a solitary species complex to evaluate the drivers of group formation. Proceedings of the National Academy of Sciences 121(12): e2312252121.
3. Mayer, A. E., T. J. McGreevy Jr., C. Brown, L. S. Ganoe, and B. D. Gerber. 2022. Transient persistence of bobcat (*Lynx rufus*) occurrence throughout a human-dominated landscape. Population Ecology 64(4): 323-335.
4. Mayer, A. E., L. S. Ganoe, C. Brown, B. D. Gerber. 2023. Diel activity structures the occurrence of a mammal community in a human-dominated landscape. Ecology and Evolution 13(11).
5. Devarajan, K., M. Fidino, Z. Farris et al. 2024 *In Review*. When the wild things are: Defining mammalian diel activity and plasticity.

ACKNOWLEDGMENTS

This project was supported by Wildlife Restoration Grants administered by the U.S. Fish and Wildlife Service, Wildlife and Sport Fish Restoration Program: Partnering to fund conservation

and connect people with nature; RI projects W23R and F19AF01093. We thank I. O'Hara, T. Kostisin, R. Healey, J. Brown, and D. Reisch for assistance in the field. We thank the following for providing land access: Audubon Society of Rhode Island, The Nature Conservancy, U.S. Fish and Wildlife Service, Providence Water, Woonsocket Water District, Richmond Rural Preservation Trust, Foster Land Trust, Westerly Land Trust, Glocester Land Trust, Coventry Land Trust, Hopkinton Land Trust, Smithfield Land Trust, South Kingstown Land Trust, East Greenwich Land Trust, Narrow River Land Trust, North Smithfield Land Trust, Burrillville Land Trust, Shelter Harbor Fire District, West Greenwich Land Trust, Johnston Land Trust, Barrington Land Conservation Trust, and the towns of Burrillville, Charlestown, Cumberland, Narragansett, North Kingstown, Glocester, Warwick, Cranston, Bristol, Barrington, and Warren.

LITERATURE CITED

Bellard, C., C. Bertelsmeier, P. Leadley, W. Thuiller, and F. Courchamp. 2012. Impacts of climate change on the future of biodiversity. Ecology Letters 15:365–377.

Brown, D. G., K. M. Johnson, T. R. Loveland, and D. M. Theobald. 2005. Rural land-use trends in the conterminous United States, 1950-2000. Ecological Applications 15:1851–1863.

Burton, A. C., E. Neilson, D. Moreira, A. Ladle, R. Steenweg, J. T. Fisher, E. Bayne, and S. Boutin. 2015. Wildlife camera trapping: A review and recommendations for linking surveys to ecological processes. Journal of Applied Ecology 52:675–685.

Clutton-Brock, T., and B. C. Sheldon. 2010. Individuals and populations: The role of long-term, individual-based studies of animals in ecology and evolutionary biology. Trends in Ecology and Evolution 25:562–573.

Crooks, K. R. 2002. Relative sensitivities of mammalian carnivores to habitat fragmentation. Conservation Biology 16:488–502.

Farris, Z. J., M. J. Kelly, S. Karpanty, A. Murphy, F. Ratelolahy, V. Andrianjakarivelo, and C. Holmes. 2017. The times they are a changin': Multi-year surveys reveal exotics replace native carnivores at a Madagascar rainforest site. Biological Conservation 206:320–328.

Gilbert, N. A, J. D. J. Clare, J. L. Stenglein, and B. Zuckerberg. 2021. Abundance estimation of unmarked animals based on camera-trap data. Conservation Biology 35:88–100.

Gompper, M. E. 2002. Top carnivores in the suburbs? Ecological and conservation issues raised by colonization of northeastern North America by coyotes. BioScience 52:185–190.

Hansen, A. J., R. L. Knight, J. M. Marzluff, S. Powell, K. Brown, P. H. Gude, and K. Jones. 2005. Effects of exurban development on biodiversity: Patterns, mechanisms, and research needs. Ecological Applications 15:1893–1905.

Hendry, H., and C. Mann. 2018. Camelot —intuitive software for camera-trap data management. Oryx 52:15–15.

Holt, R. D., and T. H. Keitt. 2000. Alternative causes for range limits: A metapopulation perspective. Ecology Letters 3:41–47.

Kucera, T. E., and R. H. Barrett. 2011. A history of camera trapping. Pages 9–26 *in* A. F. O'Connell, J. D. Nichols, and K. U. Karanth, editors. Camera Traps in Animal Ecology: Methods and Analyses. Springer, New York City, New York, USA.

Mackenzie, D. I., J. D. Nichols, J. A. Royle, K. H. Pollock, L. L. Bailey, and J. E. Hines. 2006. Occupancy Estimation and Modeling: Inferring Patterns and Dynamics of Species Occurrence. Page Occupancy Estimation and Modeling: Inferring Patterns and Dynamics of Species Occurrence: Second Edition. Elsevier, San Diego, California.

Mayer, A. E., L. S. Ganoe, C. Brown, and B. D. Gerber. 2023. Diel activity structures the occurrence of a mammal community in a human-dominated landscape. Ecology and Evolution 13.

Mayer, A., L. Ganoe, C. Brown, T. J. McGreevy, and B. Gerber. 2024. Data from: Rhode Island wildlife camera trap survey 2018 to 2023 (Version v3). Zenodo. https://doi.org/10.5281/zenodo.10610602

McRae, B. H., N. H. Schumaker, R. B. McKane, R. T. Busing, A. M. Solomon, and C. A. Burdick. 2008. A multi-model framework for simulating wildlife population response to land-use and climate change. Ecological Modelling 219:77–91.

Moss, W. E., M. W. Alldredge, and J. N. Pauli. 2016. Quantifying risk and resource use for a large carnivore in an expanding urban-wildland interface. Journal of Applied Ecology 53:371–378.

Pasquarella, V. J., J. S. Elkinton, and B. A. Bradley. 2018. Extensive gypsy moth defoliation in Southern New England characterized using Landsat satellite observations. Biological Invasions 20:3047–3053.

Prange, S., and S. D. Gehrt. 2004. Changes in mesopredator-community structure in response to urbanization. Canadian Journal of Zoology 82:1804–1817.

RIGIS, 2018*a*. Municipal & Non-Governmental Organization Conservation Lands; locCons18. Rhode Island Geographic Information System (RIGIS) Data Distribution System, URL: http://www.rigis.org, Environmental Data Center, University of Rhode Island, Kingston, Rhode Island (last date accessed: 8 January 2019).

RIGIS, 2018*b*. State Conservation Lands; staCons18. Rhode Island Geographic Information System (RIGIS) Data Distribution System, URL: http://www.rigis.org, Environmental Data

Center, University of Rhode Island, Kingston, Rhode Island (last date accessed: 8 January 2019).

Theobald, D. M. 2010. Estimating natural landscape changes from 1992 to 2030 in the conterminous US. Landscape Ecology 25:999–1011.

Wait, K. R., A. M. Ricketts, and A. A. Ahlers. 2018. Land-use change structures carnivore communities in remaining tallgrass prairie. Journal of Wildlife Management 82:1491–1502.

Wang, Y., M. L. Allen, and C. C. Wilmers. 2015. Mesopredator spatial and temporal responses to large predators and human development in the Santa Cruz Mountains of California. Biological Conservation 190:23–33.

Westgate, M. J., G. E. Likens, and D. B. Lindenmayer. 2013. Adaptive management of biological systems: A review. Biological Conservation 158:128–139.
